# The Influence of Diagnosis, Age, and Gender on Surgical Outcomes in Patients With Adult Spinal Deformity

**DOI:** 10.1177/2192568218772568

**Published:** 2018-04-29

**Authors:** Selim Ayhan, Selcen Yuksel, Vugar Nabiyev, Prashant Adhikari, Alba Villa-Casademunt, Ferran Pellise, Francisco Sanchez Perez-Grueso, Ahmet Alanay, Ibrahim Obeid, Frank Kleinstueck, Emre Acaroglu, European Spine Study Group

**Affiliations:** 1 ARTES Spine Center, Ankara, Turkey; 2 Acibadem Mehmet Ali Aydinlar University, Istanbul, Turkey; 3 Yildirim Beyazit University, Ankara, Turkey; 4 Hospital Universitari Valld’Hebron, Barcelona, Spain; 5 Hospital Universitari La Paz, Madrid, Spain; 6 Bordeaux University Hospital, Bordeaux, France; 7 Schultess Clinic, Zurich, Switzerland

**Keywords:** adult spinal deformity, age, gender, MCID, outcome, surgery, treatment, HRQOL

## Abstract

**Study Design:**

Retrospective review of prospectively collected data from a multicentric database.

**Objectives:**

To determine the clinical impact of diagnosis, age, and gender on treatment outcomes in surgically treated adult spinal deformity (ASD) patients.

**Methods:**

A total of 199 surgical patients with a minimum follow-up of 1 year were included and analyzed for baseline characteristics. Patients were separated into 2 groups based on improvement in health-related quality of life (HRQOL) parameters by minimum clinically important difference. Statistics were used to analyze the effect of diagnosis, age, and gender on outcome measurements followed by a multivariate binary logistic regression model for these results with statistical significance.

**Results:**

Age was found to affect SF-36 PCS (Short From-36 Physical Component Summary) score significantly, with an odds ratio of 1.017 (unit by unit) of improving SF-36 PCS score on multivariate analysis (*P* < .05). The breaking point in age for this effect was 37.5 years (AUC = 58.0, *P* = .05). A diagnosis of idiopathic deformity would increase the probability of improvement in Oswestry Disability Index (ODI) by a factor of 0.219 and in SF-36 PCS by 0.581 times (*P* < .05). Gender was found not to have a significant effect on any of the HRQOL scores.

**Conclusions:**

Age, along with a diagnosis of degenerative deformity, may have positive effects on the likelihood of improvement in SF-36 PCS (for age) and ODI (for diagnosis) in surgically treated patients with ASD and the breaking point of this effect may be earlier than generally anticipated. Gender does not seem to affect results. These may be important in patient counseling for the anticipated outcomes of surgery.

## Introduction

The growth of ageing population and longer life expectancy and the increased awareness in quality of life issues have made adult spinal deformity (ASD) a significant health care concern.^1^ Although the patients characteristically present with pain and disability, first-line management for symptomatic ASD without progressive neurological deficit typically involves nonoperative treatment strategies, such as physical therapy, steroid injections, nonsteroidal anti-inflammatory drugs, and narcotic analgesics, to avoid the potential morbidity of an extensive surgical intervention.^2,3^ However, for the group of patients with progressive pain, disability, and neurological problems, a decision on surgical treatment is required.^3-6^ When compared with nonoperative treatment options, surgery provides significant symptom relief and has been shown to yield better results in the overall population of patients in terms of health-related quality of life (HRQOL) outcomes, but with reported complication rates ranging from 10% to more than 80%.^3,5-9^ Hence, a significant proportion of operated patients do not improve after surgery regardless of the apparent technical success in modern ASD surgery.^5,6^

Over the past 2 decades, numerous efforts have been made to find the factors affecting the natural history, therapeutic options, and treatment outcomes in order to identify the best and proper clinical approach in ASD.^2,3,5-7,9,10^ However, the evidence is still insufficient as to whether diagnosis, gender, and/or age by itself have an influence on such circumstances in ASD.

Clinical and experimental studies have clearly indicated that women are more sensitive to pain and they are exposed to more intensive pain, more frequently, and over a longer duration than men.^11-13^ Furthermore, they experience pain in more body parts and their physical symptoms are different from those of men.^11,14^ Also, for a given pain intensity, again women display a higher level of disability and health care pursuing than do men.^15,16^ Body perception is also another issue that may be different between genders. A recent study by Pochon et al has shown that women do not differ significantly from men regarding their postoperative outcome in patients with lumbar degenerative disorders other than deformity.^13^

Age is also another important issue in patients with ASD that is closely associated and collinear with diagnosis. The presenting symptoms and the neurological status of the patients are generally different in younger adults with idiopathic deformity compared with elderly patients with degenerative pathology.^17^ Elderly patients with ASD had greater disability, greater pain intensity, and worse health status at baseline. The treatment is directed mainly by the severity of the deformity in the younger subgroup^18^; however, in the elderly, the main decisive factors for treatment are pain, poor function, and disability,^19-21^ factors that have been demonstrated to be strongly associated with several radiological parameters.^22^ When compared with their younger counterparts, the improvement was better with treatment outcome measurements, health status, and pain intensity after surgery but with more complication rates in the elderly.^3^ But in terms of self-image perception after deformity correction surgery, younger patients (<60 years old) have reported a greater change from baseline.^23^ On the other hand, Smith et al have published a research on ASD surgery comparing the best and the worst clinical outcomes, and they found that age to be an ineffective variable.^6^ So, in accordance with the current literature, the evidence is still inadequate and any cutoff point for age has still not been identified in ASD patients while making decisions.

For the evaluation of natural history and treatment outcomes, HRQOL is a multidimensional concept that includes domains related to physical, mental, emotional, and social functioning. The clinical relevance of HRQOL outcomes in ASD surgery has improved by determining thresholds necessary to gain a minimal clinically important difference (MCID).^24^ The MCID is the smallest amount of improvement on an outcome score that a patient appreciates as meaningful.^25^

From these standpoints, the authors of the current study hypothesized that age and diagnosis by themselves and/or gender may affect the surgical outcomes by means of HRQOL parameters in patients with ASD. Moreover, they tried to find out a clinically useful age cutoff point for better understanding the timing of surgical treatment in this group of patients.

## Patients and Methods

Prospectively collected data from a multicentric ASD database was reviewed retrospectively. The inclusion criteria into the database were the following: age >18 years and scoliosis >20°, or sagittal vertical axis (SVA) >5 cm, or pelvic tilt >25°, thoracic kyphosis >60°, and deformity of degenerative or idiopathic etiology. All patients were enrolled in an institutional review board–approved protocol by the respective sites. Baseline demographic data (age, gender, comorbidities, and body mass index [BMI]), HRQOL parameters (the Core Outcome Measures Index [COMI], the Oswestry Disability Index [ODI], Short-Form [SF]-36 Mental Component Summary [MCS], SF-36 Physical Component Summary [PCS], and Scoliosis Research Society-22 questionnaire [SRS-22]), and the following radiological features were included: SVA, T2-T12 kyphosis, coronal balance, major curve Cobb angle, Lordosis gap (L gap),^22^ global tilt,^26^ and T1 sagittal tilt. Patients were also stratified according to the etiology of their deformity: idiopathic, degenerative, and others.

Demographic, clinical, and radiological characteristics and HRQOL parameters (COMI, ODI, SF-36 MCS, SF-36 PCS, and SRS-22) at baseline and 1 year after the surgery were extracted. Using the distribution-based MCID calculated from the database for each HRQOL parameter,^27^ patients were dichotomized into 2 groups of “unimproved” and “improved” cohorts.^27^ If a patient who had deteriorated in terms of HRQOL or had improved, but that improvement had not reached the MCID for that HRQOL parameter at the 1-year follow-up, was categorized as unimproved.

### Statistical Analysis

The mean values of each HRQOL parameter in the “improved” and “unimproved” categories were calculated. Student’s *t* test and χ^2^ test were used to analyze the effects of diagnosis (idiopathic vs degenerative), age, and gender on outcome measurements in improved and unimproved cohorts. Type I error rate was taken as α = .05 for statistical significance.

Then, a stepwise multivariate logistic regression method was used to evaluate the impact of age and gender on HRQOL parameters. First, univariate analyses were performed to select candidate variables for logistic model (α = .25 was taken for this step). Then, significant odds ratios were obtained by multivariate analysis (Type I error rate was taken as α = .05 for statistical significance).

To determine a clinically useful preoperative age cutoff point that could predict the likelihood of a patient reaching MCID of improvement in HRQOL parameters after the surgery, we conducted receiver operating characteristic curve (ROC) analysis (*P* < .05).

Statistical analysis was performed using IBM SPSS Statistics Version 21.0 software (IBM Corp; Armonk, NY).

## Results

At the time of the preparation of this article, the multicenter database had 1050 entries, 412 of whom had been treated with surgery (posterior fusion and instrumentation in varying lengths with or without decompression procedures and with or without osteotomies, as deemed necessary by the treating surgeon), and 199 of whom had reached and completed 1-year follow-up after surgery (the lost to follow-up rate of surgical patients in this database is less than 20% at 1 year). Demographic, clinical, and radiological features and HRQOL parameters of such patients are summarized in Table 1. Although diagnosis at the time of presentation was found to be a factor with a strong association with age, it was still included in statistical analysis so as to be able to analyze its individual effects. The radiological parameters that have been evaluated were entered into the regression analysis as potential cofactors, but as none of them were found to be of significance, they were not included in the tables summarizing our results (Tables 2 and 3). When age was evaluated as a factor for comparison between improved and unimproved groups of patients as determined by MCID calculated from the database in surgically treated patients, only SF-36 PCS was found to reach statistical significance (*P* < .05; Table 2A). Regression analysis performed for SF-36 PCS in turn showed that 1 unit increment of age would increase the probability of improvement in SF-36 PCS by 1.017 times (*P* < .05; Table 2B). The breaking point in age for this effect was calculated to be 37.5 years (AUC = 0.587; *P* = .05). As for diagnosis as a factor for comparison between improved and unimproved groups of patients as determined by MCID, only ODI was found to reach statistical significance (*P* = .004), whereas SF-36 PCS was close (0.084; Table 2C). Regression analysis performed both for ODI and SF-36 PCS in turn showed that a diagnosis of idiopathic deformity would increase the probability of improvement in ODI by a factor of 0.219 and in SF-36 PCS by 0.581 times (*P* < .05; Table 2D), both suggesting that the odds for improvement by surgery are higher in degenerative deformity. On the other hand, no statistically significant difference was found between any of the HRQOL parameters for surgically “improved” and “unimproved” groups of patients for gender (Table 3).

**Table 1.** Demographic (A), Clinical (A), and Radiological (B) Features and Health-Related Quality of Life Parameters (C) of Surgically Treated Adult Spinal Deformity Patients With a Minimum of 1-Year Follow-up.

(A) Characteristic			
Age (years), mean (SD)	51.94 (19.87)		
Gender, n (%)			
Female	164 (82.4)		
Male	35 (17.6)		
Etiology, n (%)			
Degenerative	86 (43.2)		
Idiopathic	113 (56.8)		
(B) Variables	Preoperative Mean (SD)	First Year Follow-up Mean (SD)	*P*
MCCA (°)	40.85 (22.10)	20.86 (14.71)	<.001
SB (mm)	52.75 (49.37)	44.57 (37.66)	.023
LL (°)	45.59 (19.89)	49.60 (13.24)	.001
GT (°)	26.45 (17.20)	23.71 (13.87)	.004
T1-ST (°)	5.13 (3.73)	5.08 (3.35)	.877
T2-T12 kyphosis	37.68 (18.57)	45.05 (15.78)	<.001
SS (°)	33.22 (11.84)	34.39 (10.21)	.044
PT (°)	22.73 (11.13)	21.67 (10.35)	.054
(C) Variables	Preoperative Mean (SD)	First Year Follow-up Mean (SD)	*P*
ODI	40.41 (21.14)	27.92 (17.67)	<.001
SF-36 MCS	41.48 (11.78)	45.81 (11.36)	<.001
SF-36 PCS	35.94 (8.85)	41.87 (8.97)	<.001
SRS-22 Subtotal	2.86 (0.70)	3.53 (0.70)	<.001

Abbreviations: MCCA, major coronal Cobb angle; SB, sagittal balance; LL, lumbar lordosis; GT, global tilt; ST, sagittal tilt; SS, sacral slope; PT, pelvic tilt; ODI, Oswestry Disability Index; SF, Short Form; MCS, Mental Component Summary; PCS, Physical Component Summary; SRS, Scoliosis Research Society.

**Table 2.** Student’s *t* Test and Multivariate Binary Logistic Regression Model Results on the Relations Between Age (A and B) and Diagnosis (C and D) and Health-Related Quality of Life (HRQOL) Parameters.^a^

	Age							
(A) HRQOL parameter	Mean	SD	*P*					
ODI								
Unimproved	44.81	19.26	.197					
Improved	52.91	20.15						
COMI								
Unimproved	46.25	25.52	.346					
Improved	55.62	19.21						
SF-36 MCS								
Unimproved	50.80	20.56	.139					
Improved	55.40	19.35						
SF-36 PCS								
Unimproved	49.67	21.33	**.034**					
Improved	56.24	18.34						
SRS-22								
Unimproved	51.50	21.16	.678					
Improved	52.79	19.53						
							95% CI for OR	
(B)	B	SE	Wald	df	*P*	OR	Lower	Upper
Age	0.017	0.008	4.425	1	.035	1.017	1.001	1.033
Constant	−0.800	0.449	3.176	1	.075	0.450		
	Diagnosis							
(C) HRQOL Parameter	Degenerative	Idiopathic	Total	*P*				
ODI								
Unimproved	4	20	24	**.004**				
Improved	73	80	153					
Total	77	100	177					
COMI								
Unimproved	6	10	16	.689				
Improved	39	52	91					
Total	45	62	107					
SF-36 MCS								
Unimproved	30	44	74	.382				
Improved	44	49	93					
Total	74	93	167					
SF-36 PCS								
Unimproved	20	37	57	.084				
Improved	54	56	110					
Total	74	93	167					
SRS-22								
Unimproved	18	20	38	.504				
Improved	57	81	138					
Total	75	101	176					
							95% CI for OR	
(D)	B	SE	Wald	df	*P*	OR	Lower	Upper
ODI								
Diagnosis^b^	−1.518	0.571	7.063	1	.008	0.219	0.072	0.671
Constant	2.904	0.514	31.984	1	.000	18.250		
SF-36 PCS								
Diagnosis^b^	−0.579	0.337	2.954	1	.086	0.561	0.290	1.085
Constant	0.993	0.262	14.398	1	.000	2.700		

Abbreviations: ODI, Oswestry Disability Index; COMI, Core Outcome Measures Index; SF, Short Form; MCS, Mental Component Summary; PCS, Physical Component Summary; SRS, Scoliosis Research Society; OR, odds ratio; CI, confidence interval. The values in bold indicate *P* values with statistical significance (< .05).

^a^ Please note that for age only SF-36 PCS and for diagnosis only ODI and SF-36 PCS were included into the regression model as they are the only parameters with (or close to) statistical significance on Student’s *t* test.

^b^ Idiopathic.

**Table 3.** Chi-Square Test Results (A: ODI; B: COMI; C: SF-36 MCS; D: SF-36 PCS; E: SRS-22) on the Relations Between Gender and HRQOL Parameters.

		ODI MCID		
(A) *P* = .993		Unimproved	Improved	Total
Gender	Female	9	136	145
	Male	2	30	32
Total		11	166	177
		COMI MCID		
(B) *P* = .663		Unimproved	Improved	Total
Gender	Female	3	85	88
	Male	1	17	18
Total		4	102	106
		SF-36MCS MCID		
(C) *P* = .163		Unimproved	Improved	Total
Gender	Female	66	72	138
	Male	18	11	29
Total		84	83	167
		SF-36PCS MCID		
(D) *P* = .965		Unimproved	Improved	Total
Gender	Female	66	72	138
	Male	14	15	29
Total		80	87	167
		SRS-22 MCID		
(E) *P* = .191		Unimproved	Improved	Total
Gender	Female	54	90	144
	Male	16	16	32
Total		70	106	176

Abbreviations: HRQOL, health-related quality of life; ODI, Oswestry Disability Index; MCID, minimal clinically important difference; COMI, Core Outcome Measures Index; SF, Short Form; MCS, Mental Component Summary; PCS, Physical Component Summary; SRS: Scoliosis Research Society.

## Discussion

This study aimed to analyze the effects of patient age, gender, and diagnosis on the surgical treatment outcomes in ASD. A total of 199 patients who had undergone surgery and completed at least 1-year follow-up were evaluated for clinical results (HRQOL parameters) at the first year following their surgeries.

Our results indicate that the only HRQOL parameter significantly affected by patient age is the SF-36 PCS, and this effect is in the direction of an increased likelihood of improvement as the patient age increases. Furthermore, this effect becomes prominent at a relatively early age of 37 years. Although the odds ratio for this appears to be low, it needs to be noted that this ratio is calculated as a unit per unit indicator, thereby denoting a geometrical relationship between these 2 parameters. As an example, an age difference of 10 years increases the likelihood of improvement by a ratio of 1.18, and an age difference of 20 years increases it by a ratio of 1.40. This finding has several implications:

Contrary to the common belief that results of surgery should be worse in older patients, this finding suggests that, conversely, surgery increases the likelihood of a significant improvement in (at least) the SF-36 PCS significantly. This trend has also been found and reported previously,^9,21,28,29^ but this is the first study that specifically addresses this matter. Based on this, we can safely state that surgery improves the general life quality more significantly in older patients. That said, it may also be important to recognize that such a relationship exists despite the (potential) presence of a higher rate and severity of comorbidities and not necessarily smaller surgeries as the patient age gets older. Although these factors (magnitude of surgery and comorbidities) have not been investigated as cofactors in this study, it may be appropriate to summarize this finding as “the likelihood of a relevant improvement in SF-36 PCS increases with patient age regardless of the potentially higher rate and severity of comorbidities.”

As also have been demonstrated by previous studies,^9,21,28,29^ the cutoff point for the age effect is younger than that may be estimated intuitively. In this regard, it appears that the age limit after which we may call a patient “old” has a tipping point around 40 years of age. This has been demonstrated at least by one other study as well.^30^ This cutoff point makes sense as it is probably the point that signifies a shift in the reasons for surgical treatment from patients’ perspectives. We may speculate that patients younger than (approximately) 40 years of age are undergoing surgical treatment for reasons other than disability (self-image and/or back pain), whereas disability caused by the deformity (regardless of the etiology) may become the major and dominant factor after that point.

It is also interesting that the only parameter affected by age is a non–disease-specific HRQOL measure, the SF-36 PCS. Other so-called disease-specific parameters (ODI, COMI, and SRS-22) as well as the SF-36 MCS were not found to be affected; in fact, the *P* values for these are not even close to any significant relationship. In our opinion, this finding singularly attests to the complexity of ASD as a disease condition. The ASD population is such a diverse and heterogeneous population that HRQOL measures focusing on only a single facet of the potential problems (deformity for SRS-22, and disability for ODI and COMI) may fall short in acquiring insight of the entire clinical spectrum. Hence, a general HRQOL measure may be more sensitive to the effects of age. Furthermore, we may want to note that none of the HRQOL parameters that had been used in this study (or elsewhere) are specific for ASD. More work on developing a truly disease-specific HRQOL measure for ASD may be needed. On the other hand, very importantly, the reason for not finding the other HRQOL parameters being affected by age may as well be our inability to do so, due to a relatively small (albeit one of the largest so far) sample size. Similar studies with larger cohorts (and longer follow-up periods) may also be warranted. Conversely, the effect of age we see on SF-36 PCS may resemble an artefact as well, as SF-36 is a nonspecific test and may have been affected by numerous other factors. This is not particularly likely, but still it may have happened.

As for diagnosis, it is difficult to estimate its effect and importance independent from the age factor. In a cohort of ASD, degenerative patients are expected to be of older ages on average, compared those with idiopathic deformity. Therefore, results of analysis of the effects of diagnosis needs to be interpreted along with those of age. Our results have demonstrated that the odds for improvement in ODI by surgery are significantly higher in the degenerative group and probably so in SF-36 PCS but not in other HRQOL parameters. Although this may seem counterintuitive, at least one other study has also demonstrated that in degenerative patients, and especially those with higher levels of disability at presentation, surgical treatment is likely to yield higher benefit.^9^ The question that remains to be answered based on this is, “Why is this effect only significant (or, visible) for ODI and, to some extent, SF-36 PCS and not the others?” Although this study had not provided us with a direct answer to this question, we can speculate that this may be due to the inherent characteristics of the HRQOL parameters that had been investigated. Although none of the scales investigated in this study are disease specific (ie, for ASD), ODI, as it specifically addresses disability caused by degenerative conditions, may be the one that is more sensitive to the reversal of it as well.

Another important finding of this study is the lack of any effect by patient gender. On the one hand, this finding seems to be another statement of the obvious—there is no logical reason for assuming that gender should affect clinical outcomes. On the other, though, several studies have implied that gender not only is a significant factor in coping with back problems^15,16^ but may also be a significant determinant of the HRQOL in the cohort of patients with ASD.^28^ Considering these, there may be 2 explanations for our finding: (*a*) gender, albeit an important factor in disability associated with disease, may not be as important to affect the results of treatment; and (*b*) gender may have affected at least some of the parameters but our study fails to demonstrate this effect because of insufficient statistical power, especially related to the number of male patients enrolled. The authors of this study do acknowledge the relatively small number of male patients as a shortcoming. On the other hand, this number is not very small (35) and the probabilities for statistical significance (ie, *P* values) are not even close to the level of .05 (5%) for any of the parameters tested. Based on these, this finding is more likely to be a genuine lack of effect than being an artefact.

## Conclusion

This study demonstrates that patient age, along with a diagnosis of degenerative deformity, may have positive effects on the likelihood of improvement in SF-36 PCS (for age) and ODI (for diagnosis) in surgically treated patients with ASD, and the breaking point of this effect may be earlier than generally anticipated (37 years). Gender, on the other hand, does not seem to affect results. This information may be important in patient counseling for the anticipated outcomes of surgery.
